# Preparedness of non-hospital health centers to manage patients with life-threatening emergency conditions: findings from a qualitative study

**DOI:** 10.1186/s12913-020-05981-1

**Published:** 2020-12-07

**Authors:** Homayoun Sadeghi-Bazargani, Mehrdad Amir-Behghadami, Masoumeh Gholizadeh, Ali Janati, Farzad Rahmani

**Affiliations:** 1 Student Research Committee (SRC), Tabriz University of Medical Sciences, Tabriz, Iran; 2 Road Traffic Injury Research Center, Tabriz University of Medical Sciences, Tabriz, Iran; 3 Tabriz Health Services Management Research Center, Health Management and Safety Promotion Research Institute, Tabriz University of Medical Sciences, Tabriz, Iran; 4 Iranian Center of Excellence in Health Management, Department of Health Service Management, School of Management and Medical Informatics, Tabriz University of Medical Sciences, Tabriz, Iran; 5 Emergency Medicine Department, Sina Medical Research and Training Hospital, Tabriz University of Medical Sciences, Tabriz, Iran

**Keywords:** Non-hospital health centers, Preparedness, Life-threatening emergency, Qualitative study

## Abstract

**Background:**

Management of Life-threatening Emergency (LTE) patients in urban and rural areas is an important challenge, which can affect pre-hospital mortality rate. Therefore, Non-hospital Health Center (NHHC) must be prepared to manage such emergency cases that may occur in the geographic area where these centers act. The aim of this study was to explore domains related to the preparedness of NHHCs to manage LTE patients through resorting to healthcare providers’ and experts’ perspectives.

**Methods:**

A qualitative exploratory study was applied using Semi-Structured Interviews (SSIs) and Focus Group Discussions (FGDs). Prior to beginning data collection, the study and its objectives were explained to the participants and their informed consents were obtained. Then, SSIs and FGDs were conducted by two trained researchers using an interview guide, which was developed through literature review and consulting experts. In total, 12 SSIs were done with the providers at different NHHCs in Tabriz. In addition, 2 FGDs were conducted with the specialists in Emergency Medicine (EM) and Primary Health Care (PHC), and the executives of health centers, with over 5 years of work experience, and Emergency Medical Services (EMS) experts. Purposive sampling method was used in this study. All SSIs and FGDs were audio recorded and subsequently transcribed. Framework Analysis was employed to manually analyze the interview transcripts from all the SSIs and FGDs.

**Results:**

The interview transcripts analysis resulted in the emergence of 3 themes and 11 sub-themes, categorized according to Donabedian’s triple model. 5 sub-themes were related to input, including medical equipment and supplies, environmental infrastructures of the centers, emergency medicines, human resource, and protocols, guidelines and policies. 4 sub-themes were related to process, including providing clinical services, medicine storage capacity, maintenance of equipment, and management process. Finally, 2 sub-themes were related to outcome, which were patients’ satisfaction with the quality of care and improved survival of LTE patients.

**Conclusions:**

The results of this study can provide a new perspective for health managers and policy makers on how to evaluate the preparedness of NHHCs in managing LTE patients. In addition, it will be used to develop instruments to measure the preparedness of these centers.

**Supplementary Information:**

The online version contains supplementary material available at 10.1186/s12913-020-05981-1.

## Background

Management of Life-threatening Emergencies (LTE) patients in urban and rural areas is a critical challenge, which can affect the outcomes of pre-hospital mortality and morbidity. Hence, delivering basic emergency care must be taken into account at all levels of health system [1]. In recent years, Iranian Ministry of Health and Medical Education (MOHME) has begun to integrate preventive services and care in emergencies, according to injury prevention strategies of World Health Organization (WHO) [2]. Non-hospital Health Centers (NHHCs) as Community Health Centers (CHC), which consist of Primary Health Centers (PHCs) and outpatient clinics, play an important role in these efforts [3, 4].

NHHCs are responsible for delivering integrated care services to the population covered in geographically defined areas, which are mainly administered by clinical officers (*Behvarz* in Persian), nurses, and physicians [5, 6]. In Iran, the duties assigned to these centers consist of minor surgery, prevention of illness, and basic emergency care for LTE patients [7]. Since LTEs always occur unexpectedly and accidentally [8], it is expected that NHHCs be prepared to manage such emergency cases that may happen in the geographic area of these centers [9]. However, the question is whether these centers have been prepared enough to manage LTE patients or not. The results of some national and international studies have reported that these centers are unprepared in managing LTE patients [1, 7, 10–13]. Hence, preparedness of NHHCs, specifically in rural and urban slums, will play a vital role in providing basic emergency to critically injured patients and decreasing load of emergency wards of hospitals and health system costs [14].

A review of the published literature highlights a deficiency of studies on the available research. Although previous studies mostly have focused on management of LTE patients in hospitals and their preparedness, the issue has largely been overlooked in NHHCs. However, due to the fact that NHHCs cover a wider population, as the first level of health care provision, more attention must be given to managing LTE patients in these centers [15]. It is crystal clear that NHHCs will arrive at their aims only if they have accurate information about current preparation state. For this reason, using the experiences and opinions of providers and experts, the appropriate domains for preparing NHHCs in the LTEs were formulated. Domains and factors affecting the preparedness of NHHCs are multidimensional, subjective and dependent on the context. Thus, it is essential that qualitative research be designed and carried out in this regard. The purpose of this qualitative study was to explore domains associated with the preparedness of NHHCs in managing LTE patients from the perspective of providers and experts.

## Methods

The study is part of a comprehensive study conducted to develop a preparedness assessment instrument for NHHCs to manage LTE patients, and its protocol has also been published elsewhere [15].

### Study design and data collection

This was a qualitative exploratory study based on Semi-Structured Interviews (SSIs) and Focus Group Discussions (FGDs). SSIs and FGDs were conducted between September 2018 and February 2019 in accordance with the predetermined interview guide, which was developed through literature review and consulting experts **(**Additional File 1**)**. Each SSI and FGD was opened by asking with open-ended questions. Subsequent questions were asked based on the participants’ answers to the first question and also based on the interview guide. It should be noted that whenever necessary, we also ask exploratory questions such as “What do you mean by this?” “Can you give a more detailed explanation?” “How did you feel about that?” In addition, at the end of each SSI and FGD, participants were allowed to talk about any forgotten points. In FGDs, the exact timing of the sessions was coordinated with the participants via e-mail or mobile. Then, the invitation letter, including introduction of the study, its objectives, and also the time and place of the meeting, was sent to the participants through administrative automation of Tabriz University of Medical Sciences. Moreover, 1 day before the session, the time and place of the meeting were reminded to the participants. The FGDs with the experts were held in the conference room of the health service management department. Each FGD meeting was scheduled for 2–3 h, including the breaks with refreshments. All SSIs and FGDs were audio recorded and subsequently transcribed. The non-verbal signals of participants (such as the tone, silence, and emphasis) were also documented.

### Trustworthiness and rigor of the study

The SSIs and FGDs were considered by two researchers, one of whom has extensive experience in this field, so as to preserve validity and reliability. In addition, in order to ensure the validity of the findings at the end of each SSI and FGD, rigor was confirmed through participants’ validity method for establishing trustworthiness. In the end of each SSI and FGD, the participants were allowed to talk about each missed point and to confirm the validation of the presented points [16].

### Sampling and participants

The participants were recruited using purposive sampling. Purposive sampling method identifies participants with relevant experience and ensures that those who have sufficient knowledge in research scope are selected. In qualitative studies, sampling cannot be anticipated; rather it is determined based on the time of data saturation, i.e. when data collection produces no new information. In this study, SSIs and FGDs also continued until data saturation. In total, 12 SSIs were carried out with the providers who are responsible for delivering care services at NHHCs in Tabriz, northwest of Iran. The interviews were performed at the providers’ work place. In addition, 13 of the specialists took part in 2 FGDs. The people participating in the FGD meetings included specialists in Emergency Medicine (EM), Primary Health Care (PHC) and executives of health centers, with over 5 years of work experience, and Emergency Medical Services (EMS) experts.

### Data analysis

Content analysis using the “framework analysis approach” was applied to manually analyze the interview transcripts from all the SSIs and FGDs. This approach is a flexible tool that can be used in qualitative studies that seek to produce themes. It is worth noting that this approach can be used in deductive, inductive or combined types of qualitative analysis. In accordance with this approach, data analysis was performed after a five-stage procedure, which involves familiarization, developing a theoretical framework, indexing, charting, mapping and interpretation [17]. Each step of the analysis was performed by the first researcher (MAB). To improve inter-rater reliability and prevent data bias, a selection of transcripts was reviewed by another researcher (AJ). In the first stage of data analysis, the researchers immersed themselves in the data to become fully acquainted with it and to review the main themes of the data. Therefore, in order to get completely familiar with the data, the transcripts were read and re-read by MAB (familiarization). During the familiarization process, notes were made in margins of the transcripts from the main ideas that appeared to recur in the data. These recurring ideas from the familiarization process (sub-themes) were then collated into groups of similar ideas, or themes, in order to be organized into a conceptual framework, or index. The analysis of the interviews began with the Donabedin three-element framework (deductive approach). At the same time, the authors were flexible given the possibility of emerging new themes (inductive approach). MAB discussed his understanding of the transcripts with AJ to select a thematic framework for data coding (developing a theoretical framework). In addition, the main themes of the initial framework were discussed three times in the research team meetings and no new theme was added to the initial framework. We used the Donabedian model as a theoretical framework. This model emphasizes the importance of input, process, and outcome [18] “Input” stands for features of the health units and centers where care is provided. It includes such features as financial resources, human resources, equipment, and etc. “Process” points to any activity related to providing services, interaction between customers, and input of health care. “Outcome” indicates effects and consequences of any given care on health condition. Input affects processes and outcomes. Outcome reflects the effects of input and process combined. The themes from the draft framework were coded and annotated alongside the margins of the transcripts (indexing). Then, the data were summarized and placed in the appropriate theme on the theoretical framework in a chart with the help of AJ into thematic charts (charting). This step involves reducing the original data into sections of controllable text that are easily recognizable. The final step of the framework analysis comprised mapping and interpretation through which the summaries on the charts were reviewed in order to make sense of the entire data set and also the themes and sub-themes were compared with each other.

## Results

The mean age of the participants was 40 years with average work experience of 13 years (Table 1).

**Table 1:** Demographic characteristics of participants in the study

Characteristic	Qualitative variables		Number (Percent)
**Participations**	FGDs with experts	Emergency medicine	4 (16%)
		PHC specialists	2 (8%)
		Executives of health centers	5 (20%)
		EMS experts	2 (8%)
	SSIs with providers	Physicians	6 (24%)
		Nurses	6 (24%)
**Sex**	Male		20 (80%)
	Female		5 (20%)
**Age**	30–40		8 (32%)
	41–50		17 (68%)
**Work experience (Years)**	5–15		15 (60%)
	16–25		10 (40%)
**Highest level of educational degree**	Bachelor		5 (20%)
	Masters		3 (12%)
	PHD		3 (12%)
	MD, specialist		14 (56%)

The domains were categorized into 3 themes and 11 sub-themes according to the Donabedian’s triple model (Table 2).

**Table 2:** The thematic framework explaining the themes and sub-themes about preparedness of NHHCs in managing the LTE patients

Themes	Sub-themes	Issues
**Input**	**Medical equipment and supplies**	Having medical supplies and equipment for airway management, respiration, circulation and shock
		Having medical supplies and equipment for the management of specific injuries
		Having sterilization equipment
		Usability of equipment and supplies to provide prompt and uninterrupted initial interventions
	**Environmental infrastructures of centers**	Location of centers
		The layout of the internal physical space appropriately and according to the standard
	**Emergency Medicines**	Having essential emergency medicines
		The adequacy of emergency medicines to manage LTEs
		Having some medications, such as benzodiazepines and opioids
	**Human resource**	Having enough human resource
		Having an expert team including physicians and nurses for first aid and CPR when managingLTE patients
		Having a human force with practical knowledge and skill
	**Protocols, Guidelines and Policies**	Protocol for the management of acute coronary syndrome
		Protocol for managing multiple trauma patients
		Dedicated guidelines for LTEs referral
		Written guidelines for LTE potential management
		Maintenance and calibration policies of medical equipment
**Process**	**Providing Clinical Services**	Basic airway management, respiration, circulation and shock
		Oxygen
		External hemorrhage control
		Basic fracture management and splinting of external fractures
		Spine Immobilization in Multi-Trauma Patients
		Basic management of wounds and burns
	**Maintenance of Equipment**	Having a routine plan to repair the equipment
		Contract with reputable companies to routinely control equipment and ensure its proper operation
	**Medicine Storage Capacity**	Adequate monitoring of drug stocks based on the routine schedule
		Having a plan to request and supply medicines.
		Storage of medicines in better conditions
	**Management Process**	Proper referral system
		Continuing medical education
		Mortality audit of LETs or risk management in order to prevent possible errors
**Outcome**	**Pateints’ Satisfaction with the Quality of Care(Technical Quality-Services Quality)**	Effects of facilities of the centers as their service providers qualifications of patient satisfaction
	**Improved Survival of LTE Patients**	Percentage of successful CPRs
		Mortality rate
		Survival of myocardial infarction

**Fig. 1:**
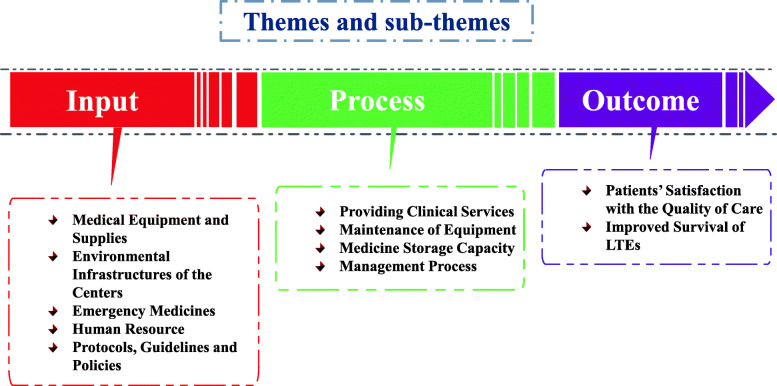
Themes and sub-themes classified based on Donabedian’s triple model

The main theme and sub-theme also are shown in Fig. 1.

### Theme1: input

#### Sub-theme 1–1: medical equipment and supplies

Medical equipment and supplies are likely to promote the preparedness of NHHCs to manage LTE patients. Hence, it is crucial that the service provider team of any given center be appropriately provided with fundamental equipment. Thus, so as to provide abrupt and immediate services, when managing LTE patients, availability and quick usability of the equipment remarkably affects preparedness of the centers.

*Majority of the experts, who participated in this study, asserted that:*

*“NHHCs should be safely and effectively equipped with fundamental equipment, based on WHO standards and guidelines, so as to manage airway, respiration, circulation, and shock*…*Not only existence of fundamental equipment is integral for preparedness and quick response to patients’ need but also quick usability of this equipment is vital for the preparedness of these centers. “FGD P*_*5,13*_*.*

#### Sub-theme 1–2: environmental infrastructures of the centers

Accessing emergency department of hospital may practically be impossible for some individuals. And, NHHCs, as primary level of health care providers when facing emergency cases, should have appropriate location and design. Hence, it is necessary that physical atmosphere of these centers be designed according to standards.

*“Evidence indicates that emergency response time and pre-hospital intervention phase are long; hence, these centers, particularly the remote ones regarding their closeness to the population covered, ought to be prepared for LTEs…interior and exterior design plays an important role in the preparedness of these centers…” FGD P*_*1,4,6,*_

#### Sub-theme 1–3: emergency medicines

NHHCs, in accordance with the number of predicted emergency cases for their covered population should have a spectrum of emergency medicines. It is because the sufficient existence of such emergency medicines in these centers can potentially play a vital role in managing LTE patients. According to the standards, accessibility and sufficiency of emergency medicines play a vital role in the preparedness of these centers.

*“When providing primary interventions for possible emergency cases having sufficient number of emergency medicines in these centers can save patients” SSI P*_*2*_*.*

#### Sub-theme 1–4: human resource

According to the participants, human resource is of significant factors affecting the way primary emergency cases are performed. Hence, it is essential to have sufficient human resource and a specialist team in accordance with the needs of the covered population of these centers so as to respond quickly when facing emergency cases. Human resource should be determined based on the needs of the region and standards. Similarly, clinical personnel with high level of knowledge and expertise can increase the preparedness of these centers.

*“Due to the possibility of sudden occurrence of LTE cases, which may occur in the geographical region of these centers, paying attention to the sufficient number of clinical personnel in accordance with the standards and having a trained specialist team are of high significance for the preparedness of these centers” SSI P*_*7*_*.*

*“Confidence of the clinical personnel in their knowledge and expertise guarantees provision of effective and timely clinical interventions…as an instance, personnel should know the difference between pink and orange Peripheral Venous Catheter (PVC) to avoid mistakes when dealing with emergency cases… If for a multi-trauma case with broken leg and torn vein and etc a pink PVC is embedded and for a Myocardial Infarction (MI) case an orange PVC is embedded, it is wrong.* …*The flow velocities of the two are different from each other (The flow rate of pink PVC is 61 ml / min and orange PVC is 343 ml / min) … So this shows the lack of knowledge of clinical personnel. FGD P*_*1,2,9*_*.*

*In my opinion, in order to assess the preparedness of non-hospital centers, we should also focus on measuring the knowledge of the personnel, especially the physicians of the centers. For instance, personnel should know that aspirin should be given to people with symptoms of chest pain, and after checking the heartbeat and blood pressure of heart attack patients, sublingual nitroglycerin tablets should be given .... I also agree with what the doctor said … Clinical staff should also be aware that for patients with diabetic ketoacidosis, crystalloid serum and insulin are the most essential components of treatment .... FGD P*_*8,10*_*.*

*“…In most cases, the skill required to do something must be combined with knowledge. For example, clinical staff should know that when intubating, the laryngoscope should be held with the right hand and open the patient’s mouth from the right…” SSI P6.*

#### Sub-theme 1–5: protocols, guidelines and policies

Lack of protocols, clinical guidelines, and standard implementation policies can lead to some complications in treatment and basic management of emergency cases. Thus, their accessibility results in updating health knowledge of the providers, particularly general physicians and nurses. On the other hand, their accessibility plays a significant role in standardizing and ensuring consistent quality of the care and hence affects the preparedness of the centers.

*“It is crystal clear that in preparedness of NHHCs, accessibility to protocols, guidelines, and policies should be noticed…these centers ought to have some protocols to manage multi-trauma cases…” FGD P*_*3,5*_*.*

*“It is integral that NHHCs surely have clinical guidelines for referral, transference, and dispatching patients. Do they have?” SSI P*_*3*_*.*

*“In my opinion, existence of a certain procedure and policies to protect and preserve medical equipment in a center stands for the quality of a center’s response” SSI P*_*11*_*.*

### Theme 2: process

#### Sub-theme 2–1: providing clinical services

Providing emergency care will vary according to the patient status, physician specialty, and distance from the nearest emergency department. NHHCs ought to have the required preparedness to provide primary emergency care. For instance, they should have the capability to manage patient’s airway, primary fractures, and the like.

*“In NHHCs, as the first level centers to provide health services, some interventions like basic management of airway, respiration, circulation and shock; controlling external bleeding; basic management of fractures; splinting external fractures; oxygen therapy; and immobility for the patients with multiple trauma should be performed at least by means of cervical collar as the first measure taken to protect neck spine of the injured…. “FGD P*_*2,7,9,10*_*.*

*“I think NHHCs are capable to provide some primary services procedures in special injuries such as basic management of cuts and burns… And if they are not able to provide such services, they should take the required measures…” SSI P*_*9*_*.*

#### Sub-theme 2–2: medicine storage capacity

Medicine storage capacity can facilitate providing safe and timely emergency care for NHHCs. Medicine storage capacity depends on its maintenance and monitoring and any incongruity in it affects preparedness of NHHCs.

*“In order to provide timely and safe emergency care, capability of NHHCs to store medicine can play a facilitating role in their preparedness… Hence, when considering preparedness of these centers, paying attention to the way emergency medicines are stored and also monitoring medicine storage are important…. “FGD P*_*11*_*.*

*“Storage conditions and monitoring medicine storage are two factors effective on medicine storage capacity of NHHCs….” SSI P*_*6*_*.*

#### Sub-theme 2–3: maintenance of equipment

Majority of the participants held that maintaining and repairing equipment in an appropriate and routine way provides the service providers with the capability of quick application of the equipment upon encountering emergency cases. Assuring availability to medical equipment and their accurate function depends on preventive maintenance and their calibration, which is required to be done by approved companies. This process, as a backup process, needs a definite and meticulous plan. As a result, accurate functioning of equipment promotes capacity and preparedness of these centers to provide emergency care.

*“Necessarily it is not required to hire a technical support team for every center to maintain and repair medical equipment; nevertheless, every center should have a routine plan…” SSI P*_*5*_*.*

*“…such centers can establish contracts with approved medical equipment companies to routinely control and calibrate their medical equipment….” SSI P*_*10*_*.*

#### Sub-theme 2–4: management process

Process includes referral of patients and risk management through Root Cause Analysis (RCA) and Failure Mode and Effects Analysis (FMEA). Not only are these processes vital for providing primary emergency care, but also they promote capacity of the center to provide care. Paying attention to these processes will end in promotion in providing care in a long run.

*“According to recent studies, it can be concluded that some emergency cases such as multi-trauma cases, myocardial infarction, and a few other cases should be referred after basic management is done…” FGD P*_*4*_*“…. Investment in referral system and accurate and timely reference of patients can promote the capacity of system to provide care and proper implementation of the referral system will improve the quality of services provided…” FGD P*_*6*_*.*

*“…any type of possible error occurring in such centers should be identified by means of RCA and FMEA, and this means quality assurance…” FGD P*_*12*_*.*

### Theme 3: outcome

#### Sub-theme 3–1: patients satisfaction with the quality of care (technical quality-service quality)

Majority of the participants expressed that the outcome affecting quality and function of such centers indicate that these centers are ready to respond to the needs of patients; hence, outcome affects preparedness of these centers.

*“Patient satisfaction with the quality of provided services depends on clinical and non-clinical aspects. In this respect, satisfaction with the quality of provided services indicates decent function of these centers.” FGD P*_*1,*_

*“…Quality of the provided care by service providers depends not only on knowledge, skills, and specialty of the service providers but also on facility standards of the centers such as accessibility of emergency medicine and primary equipment required for resuscitation as well as the way relationship is established with companions of the injured, and some other similar factors, all of which affect quality of the provided services. …. hence, NHHCs ought to endeavor to consider all the aspects, either clinical or non-clinical, affecting provided services quality…. Concentrating on these aspects is what accompanies patient satisfaction, according to which it can be claimed whether these centers are prepared to manage LTE patients or not….” FGD P*_*,2,3,6,9*_*.*

#### Sub-theme 3–2: improved survival of LTE patients

Improved survival of LTE patients depends on mortality rate, cardiac arrest survival, and percentage of successful Cardio Pulmonary Resuscitation (CPR). Mortality rate of these centers should be adjusted according to the visit tolerance of the individuals referring to such centers. Improved survival of LTE patients is an outcome, which indirectly plays a role in capacity and preparedness.

*“Mortality rate and myocardial infarction survival indicate quality level of the provided services as well as high function of these centers… if there is a low level of mortality rate in a center, it cannot be expressed that quality and function of these centers are high. It can be possible that there are few number of patients referring to the center on account of other reasons. Hence, it is essential that mortality rate be adjusted in accordance with emergency problems.” SSI P*_*9*_*.*

*“…NHHCs with successful percentages of CPR can be said to indicate better function….” SSI P*_*4*_*.*

## Discussion

In this study, which has been performed for the first time in Iran, we have identified the domains affecting NHHCs preparedness to provide primary emergency care upon facing LTE patients. The most significant domains, which affect preparedness of these centers to manage LTE patients, are summarized in input, process, and outcome. Input indicates how much the centers are capable to provide emergency care. As process and outcome are affected by input [19], it essential that, when assessing preparedness of NHHCs, we pay specific attention to inputs as the main predetermined sources for providing services. It is due to the fact that any sort of lack or defect in input can affect capacity of these centers to provide services upon facing LTE patients [20].

Input, as a practically stable concept, consists of a range of properties created through resources like human force, environmental infrastructures, equipment, and supplies [18]. Therefore, it can affect preparedness function and capacity of NHHCs [20]. Paying attention to environmental infrastructures of these centers to respond to emergency needs of the covered population is necessary. Its environment should provide the possibility of taking vital survival measures in the fastest possible time with the highest efficiency [7]. Therefore, it is highly significant to pay attention to location of centers as well as exterior and interior environment of the centers based on the standards defined for preparedness of NHHCs [21]. Proportionate to emergency needs of patients, human power skills and the distance to the closest emergency department, required medical equipment should be anticipated in order to manage LTE patients [22]. In this situation, capacity to provide primary emergency care remarkably depends on accessibility to such equipment and certainty of their accurate function [23]. Existence of emergency medicines and anticipating the least number of various medicines required for possible emergency cases according to standards and guidelines lead to survival of patients [13, 24]. Based on trauma care guidelines, emergency medicines list is defined for all health care centers [3]. Protocols and guidelines can play a vital role in updating knowledge of service providers and timely reference of patients to higher levels [25]. Not accessing to or unavailability of such protocols and guidelines will lead to a number of complications in managing LTE patients [10, 26].

People in need of emergency care may access the system in many places, including by activating the prehospital system, by visiting primary health centers and NHHCs, or by going directly to a hospital emergency unit. Providers at each level of the health system offer emergency care, whether or not they have specific training and resources available to do so effectively. Frontline emergency care may include early detection and initial resuscitation for dangerous conditions, followed by transfer to definitive care (e.g., chest evacuation, volume resuscitation, and transfusion done before transfer for surgery) or may include definitive treatment (such as the use of antibiotics for pneumonia, wound healing, or non-operative fracture management).

Potential management of LTE patients requires specific knowledge and expertise, which ought to be updated and practiced on a regular basis [12, 27]. In reality, this issue is not easy for service providers of NHHCs as they occasionally manage such conditions; hence, they may not know how they should respond appropriately even if they have required expertise as well as they have access to primary equipment [12, 28]. Due to the fact that LTEs may occur in areas where these centers are located, it is expected that such centers have required preparedness for at least basic management of LTE patients and providing pre-hospital primary care. As a result, preparedness of trained service providers of these centers to quickly respond to cases is essential [29].

Process is a collection of activities with clear objectives which are supported by resources to arrive at determined outcome [30]. Timely interventions as the main process of service such as aspirin for myocardial infarction and brain stroke [31]; considering and interpreting vital signs of patients with heart attack and prescribing nitroglycerin sublingual tablets [32]; basic management of airway, respiration, circulation, and shock [32]; providing trauma patients with pre-hospital care [33]; controlling bleeding; splinting external fractures; basic management of injuries, burns; three way dressing of vacuum wounds in chest, and other similar basic lifesaving skills for trauma [34] have saved millions of people in developed countries [34]. Process of maintaining equipment leads to ensuring the equipment availability and accurate function. This process requires planning and some logistics and also there should be some routine monitoring. In this vein, Hsia et al. considered that existence of a system to repair and maintain equipment when appraising preparedness of such centers is essential [35]. Capability to store medicines, which is vital for preparedness capacity of a treatment center and health system in providing safe and timely services, is considered regarding supplying and monitoring medicines [35]. Management process, which falls in management domain of senior managers, is helpful for better management of LTE patients and providing primary emergency care. These processes include consistent education and training, monitoring quality of reference system, auditing mortality, and risk management. Continuous education and training not only is important for patient safety but also is remarkably significant to motivate service providers, especially in rural areas. On the other hand, in order to attain development, some plans to improve accessibility to emergency cases are required. Mortality Audit and risk management provide managers with the possibility of appraising the care provided and also following up their progress in providing decent and safe care. Quality control of referral system is an important issue, in which there should be enough investment so as to buttress capacity of the centers to provide emergency care.

Outcome can indirectly leave considerable effects on preparedness capacity of NHHCs. Outcome include changes in present and future health status of patients [30, 36]. The changes may include life quality related to health and content with quality of care [30]. On the whole, patients’ satisfaction with quality of care is considered in two main aspects, i.e. technical quality and service quality. Technical quality is related to clinical aspects of health care such as proportionate relationship between the provided services and the skills of service providers [37]. Service quality requires that “services should be provided according to the needs of customers so that their expectations are met. “Normally, this issue points to non-clinical aspects and indicates patients’ experience with health care system including the relationship between patients and care providers, facility standards, and supply services [38–40].

## Conclusion

Results of the present study can provide a new perspective for authorities, planners, health managers and policy makers on how to monitor and evaluate the preparedness of NHHCs in managing LTE patients. In addition, it will be used to develop an instrument in assessing the preparedness of NHHCs to manage LTE patients. It was carried out in a way that the next step based on this study is developing an instrument by means of combining our qualitative findings with a systematic literature review of previous research. It is recommended that when appraising preparedness of NHHCs, more attention be given to input. It is because allocation of budget to these fields in low-income and developing countries is often poor. Hence, lack of fundamental input is a challenge that should be tackled. Nevertheless, essentiality of the effects of outcome should not be ignored when assessing the outcome. This study was conducted in northwestern Iran and the explored domains in this study are applicable to Iranian society. Therefore, other societies may have different domains based on the opinions of different experts.

## Supplementary Information

**Additional file 1.** Interview guide.

## Data Availability

The datasets used and/or analyzed during the current study are available from the corresponding author on reasonable request.

## Abbreviations

LTE: Life-threatening Emergency
NHHC: Non-hospital Health Center
MOHME: Ministry of Health and Medical Education
WHO: World Health Organization
CHC: Community Health Center
SSI: Semi-Structured Interview
FGD: Focus Group Discussion
EM: Emergency Medicine
PHC: Primary Health Care
EMS: Emergency Medical Services
PVC: Peripheral Venous Catheter
MI: Myocardial Infarction
RCA: Root Cause Analysis
FMEA: Failure Mode and Effects Analysis
CPR: Cardio Pulmonary Resuscitation
