# Supplementary material for: Preparedness of non-hospital health centers to manage patients with life-threatening emergency conditions: findings from a qualitative study
Source: BMC Health Serv Res. 2020 Dec 7;20:1129. doi: 10.1186/s12913-020-05981-1 (PMC7720617; doi:10.1186/s12913-020-05981-1)
Supplement: Supplementary file 1 — Additional file 1. Interview guide. [file 12913_2020_5981_MOESM1_ESM.docx]

**Interview guide**

Good time and welcome to the discussion. Thank you for taking the time to join me to talk about **Preparedness of Non-Hospital Health Centers to Manage Patients with Life-Threatening Emergency Conditions.** You have been included in this study because you are an expert in this field and play a vital role in understanding the issues pertaining to the preparedness of these centers. Therefore, I would be happy if you would provide frank answers to the questionnaire items. You are fully assured of anonymity and confidentiality of all the information provided.

**Section A: Socio-demographic data**

1. Please introduce yourself?

2. How old are you?

3. What is your education level?

4. What is your job?

5. What is your work experience?

**Section B: Interview guide questions in FGDs and SSIs with experts and health care providers**

1. If you are familiar with the concept of preparedness of non-hospital health centers when dealing with life-threatening emergencies (LTE) conditions, in accordance with the Iranian health system, what characteristics should these centers have in order to be able to manage these situations in a timely manner? please explain.
2. Based on the current capacity of the health care system in Iran and the tasks assigned to these centers, what do you think should be considered to assess the capacity of non-hospital health centers for the basic management of LTE conditions? please explain.
3. In assessing the preparedness of non-hospital health centers, is it enough to just pay attention to its availability or adequacy? Or it is necessary to pay attention to other aspects as well? please explain.
4. What can be the impact factors in the preparedness of non - hospital health centers that deal with the problems caused by the lack of initial management of LTE conditions? please explain.
5. In general, how can the availability of basic logistics affect the preparedness of non-hospital centers? Please explain
